# Supplementary material for: Trends in US pediatric mental health clinical trials: An analysis of ClinicalTrials.gov from 2007–2018
Source: PLoS One. 2021 Apr 1;16(4):e0248898. doi: 10.1371/journal.pone.0248898 (PMC8016324; doi:10.1371/journal.pone.0248898)
Supplement: S1 Table — (DOCX) [file pone.0248898.s001.docx]

**S1 Table. Changes to the initial protocol.**

| **Changes to the original protocol** | **Rationale for changes** |
| --- | --- |
| (1) We limited our analysis to only United States trials in our revised protocol. | Initially we included United States and international trials together in our sample to capture the full range of interventional mental health studies in the ClinicalTrials.gov registry (n=1662). This practice has been used in several previous analyses of the ClinicalTrials.gov registry [10, 13, 25]. However, we later determined that including international trials likely introduced significant bias into our sample, as trials from different countries are subject to different laws and incentives for reporting. It would be difficult to determine whether trends in the data were due to regional differences in clinical research or regional differences in trial registration with ClinicalTrials.gov. Therefore, as previously published with this dataset [17], we decided to keep our study limited to US trials, which we think leads to more interpretable results with fewer sources of confounding. We cite the need for analysis of international mental health trials as an area for further study in our Discussion section. |
| (2) We combined the ClinicalTrials.gov categories 'NIH' and 'US Fed' to create a new funder category 'US Govt.' | Initially we had excluded 'US Fed'-funded studies from our analysis, as they comprise only 3.5% of studies in the ClinicalTrials.gov registry and were excluded for this reason in other analyses of the ClinicalTrials.gov registry [13]. However, we later chose to include both 'NIH' and 'US Fed' studies to form a new funder category called 'US Govt,' as has been done in other analyses of the database to better capture changes in US government-funded trials [16, 17]. |
| (3) We grouped Phase 1/2 trials with Phase 2 trials into a new combined category, and we grouped Phase 2/3 trials with Phase 3 trials into a new combined category. | Given that Phase 1/2 and Phase 2/3 trials did not clearly fit an FDA-defined phase, our initial protocol grouped these trials into the category ‘Not Applicable’ (NA). However, given Phase 1/2 trials reach Phase 2 status and Phase 2/3 trials reach Phase 3 status, we later chose to group these trials together, respectively, rather than with NA. Therefore, in our final analysis, our new phase categories are Phase 1, Phase 1/2 - 2, Phase 2/3 - 3, Phase 4, and NA. |
| (4) We included two new references (Arnow et al., 2019 and Wortzel et al., 2020) [16, 17]. | The manuscript by Arnow and colleagues was published after we wrote our protocol and completed our initial analysis. We have added it to our revised protocol because this paper was fundamental to why we subsequently adjusted several aspects of our analysis. We also added the manuscript by Wortzel and colleagues to our revised protocol because the suggestions we received from reviewers for this study were instrumental to the changes that were made in this protocol. |
| (5) We changed the name of two of the treatment categories to provide clarity. | Two of the terms used to label trial treatments were considered potentially confusing to readers. The term ‘Stimulation’, originally used to denote trials testing electroconvulsive therapy, deep brain stimulation, and transcranial magnetic stimulation, was changed to ‘Stimulation’. Trials that did not fit into the treatment categories of ‘Pharmacotherapy’, ‘Psychotherapy’, or ‘Stimulation’ were originally labeled ‘Alternative’. However, given that “alternative” can have a connotation of meaning “alternative to conventional biomedicine”, this term was changed to ‘Non-Psycho/Pharmacotherapy’. |
| (6) We changed the alpha threshold from 0.01 to 0.005, in accordance with guidelines for more stringent statistical reporting | In the original analysis, an alpha threshold of 0.01 was used, meaning that the probability of mistakenly rejecting a null hypothesis was 0.01. However, in accordance with advances in more stringent statistical reporting [26], a threshold of α=0.005 was implemented in the revision of this analysis. |
