# Supplementary material for: Trends in US pediatric mental health clinical trials: An analysis of ClinicalTrials.gov from 2007–2018
Source: PLoS One. 2021 Apr 1;16(4):e0248898. doi: 10.1371/journal.pone.0248898 (PMC8016324; doi:10.1371/journal.pone.0248898)
Supplement: S2 Table — (DOCX) [file pone.0248898.s002.docx]

**S2 Table. Non-*DSM-5* subcategorization.**

| **Non-*DSM-5* Subcategories** | **Number of Trials (% of the Non-*DSM-5* category)** |
| --- | --- |
| Executive Function (Outside of Peer Contexts) | 45 (21.8) |
| Social Cognition, Bullying, and Peer Problems | 27 (13.1) |
| Stress/Wellness | 26 (12.6) |
| Suicide/Self-Harm | 25 (12.1) |
| Parenting/Attachment | 15 (7.3) |
| Abuse/Maltreatment | 11 (5.3) |
| Pain | 9 (4.4) |
| Other | 37 (18.0) |
| Not Enough Information Provided to Determine | 20 (9.7) |

Legend: Trials were labeled with as many subcategories as were relevant, and consequently the total number and percentage of Non-*DSM-5* trials sum to greater than n=206 or 100%.
