# Supplementary material for: Trends in US pediatric mental health clinical trials: An analysis of ClinicalTrials.gov from 2007–2018
Source: PLoS One. 2021 Apr 1;16(4):e0248898. doi: 10.1371/journal.pone.0248898 (PMC8016324; doi:10.1371/journal.pone.0248898)
Supplement: S1 File — (DOCX) [file pone.0248898.s003.docx]

**S1 File.**

**Study Protocol**

1. **Background:**

The ClinicalTrials.gov database was first released in 2000 to facilitate the monitoring and study of clinical trials. Reporting of results into the database was first required after the passing of the FDA Amendments Act of 2007 (FDAAA). The passing of the Final Rule clarified which trials were obligated to register and submit results, and went into effect in January 2017.

Several studies have examined this database to answer questions about the mental health clinical research enterprise [1-3]. These studies have shown that what is studied in mental health has changed significantly over the past decade, which has implications for research funding and trends in research interests. This analysis has not been performed in pediatric mental health clinical research within the ClinicalTrials.gov database. We wish to perform an analysis of how the field has grown, how the disorders and treatments studied have changed, as well as how funding for pediatric mental health research has changed. We also aim to identify how disorders and treatments studied differed by funder type, as well as how the treatments studied differed by disorder categories.

1. **Study Objectives:**

Primary Objective: To assess the funding, trial design, disease focus, and treatments studied in pediatric mental health trials in the ClinicalTrials.gov database between 2007 and 2018.

- 1. Research Questions:

Research Question #1: How many new United States trials in pediatric mental health are submitted each year, and is this quantity consistently increasing or decreasing?

Research Question #2: Is there a difference in the general design of US pediatric mental health clinical trials over time (e.g. use of blinding, randomization, multiple facilities)?

Research Question #3: What are the disorders and interventions that are the focus of US pediatric mental health related trials, and how is this different depending on who is funding the trial?

Research Question #4: How do the treatments studied differ by disorder category?

Specific Aims:

Specific Aim #1: To identify US pediatric mental health trials within the ClinicalTrials.gov database and determine their growth pattern and changes in trial characteristics (e.g. use of blinding, randomization, multiple facilities).

Specific Aim #2: To use trial records to identify which pediatric mental health disorders and interventions are studied within the registry, and to identify any differences between Industry, NIH / U.S. Fed, and Other funders of US trials.

Specific Aim #3: To use trial records to identify how funding source and interventions studied differ by the *DSM*-5 disorder categories studied.

- 1. Hypotheses:

Hypothesis #1: The number of US pediatric mental health trials has not grown over time.

Hypothesis #2: The number and proportion of trials studying psychotherapy, pharmacotherapy, interventional, alternative treatments in US pediatric mental health trials has not changed significantly over time.

Hypothesis #3: US pediatric mental health trials have disproportionately focused on autism spectrum disorder and attention-deficit/hyperactivity disorder, and there will be no differences in the disorders studied among the different funding categories.

Hypothesis #4: The number and proportion of US pediatric trials studying Non-DSM conditions increased, as was seen in the larger mental health trials portfolio, and this increase will coincide with the adoption of the NIMH Research Domain Criteria (RDoC) in 2012-2013 [4].

Hypothesis #5: Pharmacotherapies will be studied significantly less than psychotherapies, interventional therapies, and alternative therapies across all conditions in US pediatric mental health trials.

Hypothesis #6: The proportion of pharmacotherapies, psychotherapies, interventional therapies, and alternative therapies studied in US pediatric mental health trials will not differ significantly by funder type.

1. **Methods**

3.1 Study Type and Design:

We will use a retrospective, cross-sectional analysis of all US clinical trials in the ClinicalTrials.gov database. Given that we wish to assess the present state of pediatric clinical trials in mental health trials, we believe our cross-sectional approach is appropriate and will include both historically completed trials and those that were still active at the time the dataset was downloaded.

3.2 Data Source:

We will access records from ClinicalTrials.gov using the Aggregate Analysis of ClinicalTrials.gov (AACT), a relational database of publicly available ClinicalTrials.gov data.

3.3 Study Population:

We focus our analysis on US mental health trials. These we will identify by manually reviewing all medical subject headings (MeSH) and Disease Condition Terms contained in the entire ClinicalTrials.gov database. These are applied using an algorithm by the National Library of Medicine (NLM), which analyzes text in the trial record to determine the relevant MeSH terms associated with the trial. We will identify all MeSH terms that are related to mental health using review by a team of psychiatrists. To reduce the risk of misclassification bias, we will manually review all trial records using their official title and study description to confirm if they are relevant to mental health. The MeSH terms used for this analysis have been previously published [2]. Trials will be identified as studying pediatric populations if the trial description discussed studying ‘children’, ‘adolescents’, or patients ≤18 years old.

We include trials submitted from October 1, 2007. We chose this date due to the passing of the FDAAA on September 27, 2007. We downloaded records on April 30, 2018. We will exclude any trial which is not interventional (i.e. observational studies, which can also be registered in the ClinicalTrials.gov database).

3.4 Variable Definition:

We will analyze the following 11 variables, as clarified in the table below: date of submission, primary objective of the intervention, trial phase, number of arms, use of blinding, use of randomization, oversight by a data monitoring committee (DMC), number of sites, funding source (i.e. lead sponsor and collaborator class to determine all funders involved), treatment types, and disorder categories studied, With the exception of mental disorder focus and treatment types studied, all variables will be downloaded directly from the AACT.

| **Domain** | **Variable** | **Original Values** |
| --- | --- | --- |
| Covariate | Study first submitted date | 2007-2018 |
| Covariate | Primary objective | 0: Treatment 1: Basic Science 2: Prevention 3: Device feasibility 4: Diagnostic 5: Health Services Research 6: Screening 7: Supportive Care 8: Other |
| Covariate | Phase | 0: Early Phase 1 1: Phase 1 2: Phase 1/Phase 2 3: Phase 2 4: Phase 2/Phase 3 5: Phase 3 6: Phase 4 7: N/A |
| Covariate | Number of Arms | 1-28 |
| Covariate | Masking | 0: None 1: Single 2: Double |
| Covariate | Use of randomization | 0: No randomization 1: Randomization |
| Covariate | Oversight by a DMC | 0: No 1: Yes |
| Covariate | Number of Sites | 1-3511 |
| Covariate | Funding Source (Sponsor) | 0: Industry 1: NIH 2: U.S. Fed 3: Other |
| Covariate | Treatment Type | 0: Alternative  1: Interventional  2: Pharmacotherapy  3: Psychotherapy |
| Covariate | DSM Category | See Table Below |

For analysis of primary objective, a category ‘Other’ will be generated by combining the category Other in ClinicalTrials.gov with the categories Diagnostic, Health Services Research, Screening, and Basic Science

For analysis of phase, we will combine Early Phase 1 and Phase 1 into ‘Phase 1.’ Phase 1/2 – 2’ was generated by grouping the ClinicalTrials.gov categories Phase 1/2 and Phase 2. ‘Phase 2/3 – 3’ was generated by grouping the ClinicalTrials.gov categories Phase 2/3 and Phase 3. ‘Phase 4’ and ‘Not Applicable’ were taken directly from these corresponding categories in ClinicalTrials.gov.

For analysis of Funder, we combined U.S. Fed with NIH into a new category ‘US Govt,’ as has been done in prior analyses of the ClinicalTrials.gov database [1].

To define our Diagnostic and Statistical Manual (DSM) categories, we will use *DSM-5* Section II Diagnostic Criteria and Codes designations below, and any disorders not defined within *DSM-5* will be assigned the label “Non-DSM” [5].

| **Section II Diagnostic Criteria and Codes** | **Disorders Included** |
| --- | --- |
| Neurodevelopmental Disorders | Global Developmental Delay, Unspecified Intellectual Disability, Communication Disorders, Autism Spectrum Disorder, Attention Deficit/Hyperactivity Disorder, Specific Learning Disorder, Motor Disorders, Tic Disorders |
| Schizophrenia Spectrum and Other Psychotic Disorders | Delusional Disorder, Brief Psychotic Disorder, Schizophreniform Disorder, Schizophrenia, Schizoaffective Disorder, Substance/Medication-Induced Psychotic Disorder, Psychosis NOS, Catatonia |
| Bipolar and Related Disorders | Manic Episode, Hypomanic Episode, Major Depressive Episode, Bipolar I Disorder, Bipolar II Disorder, Cyclothymic Disorder, Substance-Induced Bipolar Disorder |
| Depressive Disorders | Major Depressive Disorder, Persistent Depressive Disorder, Premenstral Dysphoric Disorder, Substance/Medication-Induced Depressive Disorder, Depressive Disorder Due to Another Medical Condition, Unspecified Depressive Disorder |
| Anxiety Disorders | Selective Mutism, Specific Phobia, Social Anxiety Disorder, Panic Disorder, Agoraphobia, Generalized Anxiety Disorder, Substance/Medication-Induced Anxiety Disorder, Anxiety Disorder Due to Another Medical Condition, Unspecified Anxiety Disorder |
| Obsessive-Compulsive and Related Disorders | Body Dysmorphic Disorder, Hoarding Disorder, Trichotillomania, Excoriation, Substance/Medication-Induced Obsessive-Compulsive and Related Disorder, Obsessive-Compulsive and Related Disorder Due to Another Medical Condition, Other Specified Obsessive-Compulsive and Related Disorder, Unspecified Obsessive-Compulsive and Related Disorder |
| Trauma- and Stressor-Related Disorders | Disinhibited Social Engagement Disorder, Posttraumatic Stress Disorder, Acute Stress Disorder, Adjustment Disorders, Other Specified Trauma- and Stressor-Related Disorders, Unspecified Trauma- and Stressor-Related Disorders |
| Dissociative Disorders | Dissociative Amnesia, Depersonalization/Derealization Disorder, Other Specified Dissociative Disorders, Unspecified Dissociative Disorder |
| Somatic Symptom and Related Disorders | Illness Anxiety Disorder, Conversion Disorder, Factitious Disorder, Factitious Disorder Imposed on Another, Other Specified Somatic Symptom and Related Disorder, Unspecified Somatic Symptom and Related Disorder |
| Feeding and Eating Disorders | Rumination Disorder, Avoidant/Restrictive Food Intake Disorder, Anorexia Nervosa, Bulimia Nervosa, Binge-Eating Disorder, Other Specified Feeding or Eating Disorder, Unspecified Feeding or Eating Disorder |
| Sleep Disorders | Breathing-Related Sleep Disorder and Sleep-Wake Disorders clustered together: Hypersomnolence Disorder, Narcolepsy, Central Sleep Apnea, Sleep-Related Hypoventilation, Circadian Rhythm Sleep-Wake Disorders |
| Parasomnias | Nightmare Disorder, Rapid Eye Movement Sleep Behavior Disorder, Restless Legs Syndrome, Substance/Medication-Induced Sleep Disorder, Other Specified Insomnia Disorder, Unspecified Insomnia Disorder, Other Specified Hypersomnolence Disorder, Unspecified Hypersomnolence Disorder, Other Specified Sleep-Wake Disorder, Unspecified Sleep-Wake Disorder |
| Sexual Dysfunctions | Erectile Disorder, Female Orgasmic Disorder, Female Sexual Interest/Arousal Disorder, Genito-Pelvic Pain/Penetration Disorder, Male Hypoactive Sexual Desire Disorder, Premature (Early) Ejaculation, Substance/Medication-Induced Sexual Dysfunction, Other Specified Sexual Dysfunction, Unspecified Sexual Dysfunction |
| Gender Dysphoria | Other Specified Gender Dysphoria, Unspecified Gender Dysphoria |
| Disruptive, Impulse-Control, and Conduct Disorders | Intermittent Explosive Disorder, Conduct Disorder, Antisocial Personality Disorder, Pyromania, Kleptomania, Other Specified Disruptive, Impulse-Control, and Conduct Disorder, Unspecified Disruptive, Impulse Control, and Conduct Disorder |
| Substance-Related and Addictive Disorders | Substance-Related Disorders, Alcohol-Related Disorders, Caffeine-Related Disorders, Cannabis-Related Disorders, Hallucinogen-Related Disorders, Inhalant-Related Disorders, Opioid-Related Disorders, Sedative, Hypnotic-, or Anxiolytic-Related Disorders, Stimulant-Related Disorders, Tobacco-Related Disorders, Other (or Unknown) Substance-Related Disorders |
| Neurocognitive Disorders | Delirium, Other Specified Delirium, Unspecified Delirium. Of note, Major and Minor Neurocognitive Disorders, such as Alzheimer’s Disease and Traumatic Brain Injury were not included in this analysis, as these conditions were found to have too much overlap with the neurology literature. |
| Personality Disorders | Cluster A Personality Disorders, Cluster B Personality Disorders B, Cluster C Personality Disorders, and Other Personality Disorders |
| Paraphilic Disorders | Exhibitionistic Disorder, Frotteuristic Disorder, Sexual Masochism Disorder, Sexual Sadism Disorder, Pedophilic Disorder, Fetishistic Disorder, Transvestic Disorder, Other Specified Paraphilic Disorder, Unspecified Paraphilic Disorder |
| Non-DSM | Conditions that did not fall into any of the above categories (e.g. suicide, wellness, burnout). |

3.5 Sample Size:

A previous analysis of ClinicalTrials.gov from 2007-2018 identified 6,302 US interventional mental health trials, and slightly over 1000 were identified as involving pediatric populations [2]. We expect that most of these studies exclusively studied pediatric populations and will meet criteria for inclusion in this analysis. Due to the large number of studies within this sample, for our main outcome, using chi-square analysis, using 1000 trials, 19 degrees of freedom (the maximum for any of our covariates), an α of 0.005, and a small effect size (w = 0.2), our test power is ~1.00. This alpha was chosen to be in accordance with advances in more stringent statistical reporting [6].

3.6 Statistical Analysis Plan:

[3.6.1 – Data Management]:

All data will be accessed by download from the AACT SQL database and stored on a research laptop. All data cleaning will be conducted using R version 3.5.0. We will export trial records into excel tables for manual review for mental health disorder assignment. Otherwise all labeling will be done in R.

[3.6.2 – Descriptive Statistics]:

All tests will be two-sided with α = 0.005. We will use Chi-square tests for all categorical data. We will quantify annual change over time using the average annual growth rate (AAGR) and the compound annual growth rate (CAGR). The formula for AAGR is:

$$AAGR=\frac{GR_{1}+GR_{2}+\ldots+GR_{n}}{N}$$

Where N = number of years, GR_A_ = growth rate in year 1, GR_b_ = growth rate in year 2, and GR_n_ = growth rate in the final year.

The formula for CAGR is:

$${CAGR=\left( \frac{Quantity in final year}{\left( Quantity in first year \right)} \right)}^{\frac{1}{\# of years}}-1$$

We will assess for statistical significance of monotonic trends over time using the Mann-Kendall test. We will also create a binary time variable to assess for changes between an early and late period (defined using the mid-point between our sample period).

1. **Study Limitations**

4.1 Data Source:

While the ClinicalTrials.gov repository is one of the largest in the world, it is not complete, and many trials, especially those outside of the United States, are registered in other registries or are simply not registered. Many USA trials are not required to be registered [7]. In addition to the subset of trials not present in the database, a minority of trials in the database do not have complete records and are missing specific elements (e.g. do not report funder). This may limit the accuracy of our findings and also may limit the generalizability of our findings beyond the ClinicalTrials.gov database.

4.2 Study Design:

Because *DSM*-5 disorders will be assigned by manual review by a team of psychiatrists, it is possible that some trials could be labeled differently if observed by a different psychiatrist. Our team will collectively reviewed a set of 250 trials to allow synchronization of definitions and confusing cases, and unclear scenarios will be reviewed by the lead authors. However, it is still possible that some labeling decisions may shift the representation of different disorders in the record, which could be more impactful for disorders which are infrequent for the database.

4.3 Statistical Analyses:

Due to our large cohort, we suspect that some results may reach our threshold for significance, α = 0.005 due to chance. Because this is an exploratory analysis meant to identify important trends within our field, we do not adjust for multiple hypothesis testing, which could also further limit the true statistical significance of our results.

1. **Discussion**

This study will potentially identify major trends in the production of United States pediatric mental health clinical research. Because much of pediatric mental health funding, research, and practice are targeted to specific disorders, we hope to provide a nuanced look at the distribution of various *DSM*-5 disorders among pediatric mental health’s research portfolio. Further dividing that data by funding source will provide a closer look at which organizations are driving the clinical research for specific disorders and treatments, and the over- or under-representation of various disorders should be discussed with key stakeholders to determine if any changes are warranted.

1. **References**

1. Arnow KD, King AC, Wagner TH. Characteristics of mental health trials registered in ClinicalTrials.gov. Psychiatry Res. 2019;281:112552. Epub 2019/10/19. doi: 10.1016/j.psychres.2019.112552. PubMed PMID: 31627072.

2. Wortzel JR, Turner BE, Weeks BT, Fragassi C, Ramos V, Truong T, et al. Trends in mental health clinical research: Characterizing the ClinicalTrials.gov registry from 2007–2018. PLOS ONE. 2020;15(6):e0233996. doi: 10.1371/journal.pone.0233996.

3. Anand V, Ghosh S, Anand A. Is There a Priority Shift in Mental Health Clinical Trials? Studies in health technology and informatics. 2017;245:280-4. Epub 2018/01/04. PubMed PMID: 29295099.

4. Insel T. Post by former NIMH director Thomas Insel: Research domain criteria — RDoC. In: Health HIoM, editor. Bethesda, MD2012.

5. Diagnostic and Statistical Manual of Mental Disorders. DSM Library: American Psychiatric Association; 2013.

6. Benjamin DJ, Berger JO, Johannesson M, Nosek BA, Wagenmakers EJ, Berk R, et al. Redefine statistical significance. Nature Human Behaviour. 2018;2(1):6-10. doi: 10.1038/s41562-017-0189-z.

7. Tse T, Fain KM, Zarin DA. How to avoid common problems when using ClinicalTrials.gov in research: 10 issues to consider. Bmj. 2018;361:k1452. Epub 2018/05/29. doi: 10.1136/bmj.k1452. PubMed PMID: 29802130; PubMed Central PMCID: PMCPMC5968400 declaration of interests and declare the following interests: none.
